# Vascular Imaging With ^18^F-Fluorodeoxyglucose Positron Emission Tomography Is Influenced by Hypoxia

**DOI:** 10.1016/j.jacc.2017.01.050

**Published:** 2017-04-11

**Authors:** Francis R. Joshi, Roido Manavaki, Tim D. Fryer, Nichola L. Figg, Judith C. Sluimer, Franklin I. Aigbirhio, Anthony P. Davenport, Peter J. Kirkpatrick, Elizabeth A. Warburton, James H.F. Rudd

Vascular imaging with ^18^F-fluorodeoxyglucose positron emission tomography (^18^FDG-PET) provides a noninvasive surrogate of inflammation and has been used to test novel drug treatments for atherosclerosis (1). Hypoxia exists in atherosclerosis (2) and may contribute to the measured FDG signal (3). ^18^F-fluoromisonidazole (FMISO) PET can quantify hypoxia in tumors; it has not been applied to human atherosclerosis.

Our hypotheses were: 1) carotid plaques that cause transient ischemic attack (TIA) or stroke are more hypoxic than contralateral asymptomatic plaques; 2) carotid FDG and FMISO PET signals are positively correlated; and 3) carotid FMISO PET signal positively correlates with plaque hypoxia-inducible factor-1α (HIF-1α) staining.

Sixteen participants with carotid atherosclerosis (mean age 70 ± 7 years; 69% male; 8 recently symptomatic) underwent computed tomography (CT) angiography and PET/CT imaging with FDG (250 MBq) and FMISO (300 MBq). FMISO PET imaging occurred within 2 ± 1 days of FDG PET imaging, and symptomatic individuals underwent imaging 16 ± 11 days after TIA/stroke.

Dynamic imaging of FMISO (120 to 180 min after tracer injection) allowed estimation of both the mean K_i_ (net influx rate constant) and mean of maximum target-to-background ratio (mean max TBR) (derived from the final 3 acquisition frames 165 to 180 min post-injection). Carotid endarterectomy was performed a median of 6 days (range: 3 to 15 days) after imaging. Eleven plaques were processed for immunohistochemistry: mean percentage area CD68 and α-smooth muscle actin staining; mean CD31 staining/mm^2^; and numbers of HIF-1α nuclei.

Mean max TBR of FMISO was significantly greater in symptomatic plaques than that in contralateral lesions (1.11 ± 0.07 vs. 1.05 ± 0.06; p <0.05) (Figure 1). Mean K_i_ in symptomatic plaques was also significantly higher than that in asymptomatic plaques (3.6 × 10^−4^ ± 2.9 × 10^−4^ min^−1^ vs. 1.6 × 10^−4^ ± 1.6 × 10^−4^ min^−1^; p = 0.03). FMISO uptake correlated positively with FDG (TBR: r = 0.51; p < 0.01 and mean K_i_: r = 0.43; p = 0.02). FDG uptake and plaque macrophage content were strongly related (r = 0.67; p = 0.02). There was a trend to a positive correlation between FMISO and plaque macrophages (r = 0.52; p = 0.10). There was no relationship between FMISO uptake and risk factors for atherosclerosis, plaque smooth muscle content, or numbers of positively stained nuclei for HIF-1α. There was a trend to a negative correlation between the FMISO signal and CD31 staining (r = −0.62; p = 0.08).

In summary, our first and second hypotheses were proven: 1) culprit carotid plaques after TIA or stroke are more hypoxic than asymptomatic lesions, and 2) plaque hypoxia makes a significant contribution to carotid artery FDG PET signals. The third hypothesis, linking imaging and a tissue marker of hypoxia, was not proven.

Hypoxia has been linked with adverse features of plaque biology, including inflammation and intraplaque hemorrhage (2). Furthermore, inflammatory stimuli increase glycolytic flux in macrophages, and this effect is amplified in hypoxic conditions (4). We now suggest that hypoxia is more common in symptomatic lesions.

The robust correlation between FDG and FMISO signals suggests that hypoxia contributes to the FDG signal in FDG PET studies of atherosclerosis. In a pilot study using a related PET hypoxia tracer, HX-4, van der Valk et al. (5) drew similar conclusions (r = 0.75; p = 0.03). We extended this result using a better validated tracer and a larger patient cohort; the congruence of both static and dynamic measures of hypoxia further supports our conclusions.

The principal study limitation was the sample size. Caution should be used in the interpretation of data other than those regarding our a priori hypotheses; no adjustment was made for multiple observations within individuals. Tissue fixation methods might have adversely affected HIF-1α immunohistochemistry, although cancer studies also reported conflicting results with respect to correlations between hypoxia imaging and HIF-1α. Nevertheless, we hope these data stimulate further study into hypoxia and markers of plaque destabilization.

We reported the first prospective human study to quantify hypoxia in atherosclerosis using the validated PET tracer, FMISO. Symptomatic carotid plaques were more hypoxic than asymptomatic lesions, perhaps identifying a novel target for drug therapy. The correlation between FDG and FMISO suggested that hypoxia contributes to the FDG signal in atherosclerosis PET studies.

## Figures and Tables

**Figure 1:**
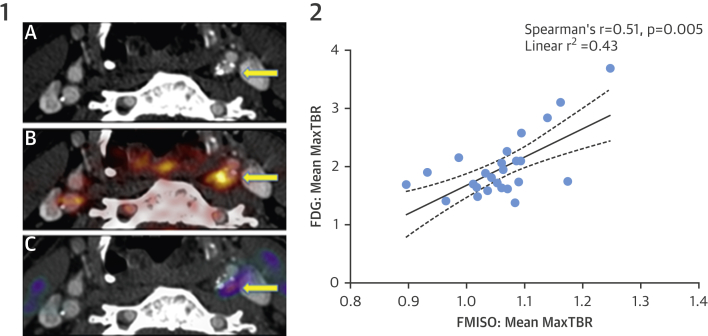
Imaging of Hypoxia in Carotid Atherosclerosis **1)** Positron emission tomography/computed tomography (PET/CT) imaging of a culprit carotid stenosis after stroke. **(A)** CT angiography. Stenosis in the left internal carotid artery **(arrow). (B)** Fused ^18^F-fluorodeoxyglucose (FDG) PET/CT. Intense uptake in the culprit lesion **(arrow)**. **(C)** Fused ^18^F-fluoromisonidazole (FMISO) PET/CT. Corresponding uptake is indicative of intraplaque hypoxia **(arrow)**. **2)** Carotid uptake of FDG and FMISO are positively correlated. Mean max TBR = mean of maximum target-to-background ratio.

## References

[1] Tarkin J.M., Joshi F.R., Rudd J.H.F. (2014). PET imaging of inflammation in atherosclerosis. Nat Rev Cardiol.

[2] Sluimer J.C., Gasc J.-M., van Wanroij J.L. (2008). Hypoxia, hypoxia-inducible transcription factor, and macrophages in human atherosclerotic plaques are correlated with intraplaque angiogenesis. J Am Coll Cardiol.

[3] Folco E.J., Sheikine Y., Rocha V.Z. (2011). Hypoxia but not inflammation augments glucose uptake in human macrophages. J Am Coll Cardiol.

[4] Tawakol A., Singh P., Mojena M. (2015). HIF-1α and PFKFB3 mediate a tight relationship between proinflammatory activation and anerobic metabolism in atherosclerotic macrophages. Arterioscler Thromb Vasc Biol.

[5] van der Valk F.M., Sluimer J.C., Vöö S.A. (2015). In vivo imaging of hypoxia in atherosclerotic plaques in humans. J Am Coll Cardiol Img.

